# Clinical Management of Respiratory Adverse Events Associated With Amikacin Liposome Inhalation Suspension: Results From a Patient Survey

**DOI:** 10.1093/ofid/ofaa079

**Published:** 2020-03-02

**Authors:** Colin Swenson, Nicole C Lapinel, Juzar Ali

**Affiliations:** 1 Division of Pulmonary, Allergy, Critical Care, and Sleep Medicine, Emory University, Atlanta, Georgia, USA; 2 Section of Pulmonary/Critical Care Medicine & Allergy/Immunology, Louisiana State University Health Sciences Center, New Orleans, Louisiana, USA

**Keywords:** ALIS, amikacin, MAC lung disease, survey, tolerability

## Abstract

Patients with *Mycobacterium avium* complex lung disease treated with amikacin liposome inhalation suspension (ALIS) at 2 clinics in the United States were surveyed to assess the frequency and management of ALIS-associated respiratory adverse events. Most respondents experienced these events, but management through physician-guided measures (eg, bronchodilator use, oral rinses, and/or temporary dosing adjustments) resulted in symptomatic improvement.

Inhaled antibiotics serve an important role in the treatment of lung infections, particularly in patients with chronic lung diseases that share similar infection pathways and disease processes (eg, structural airway diseases including cystic fibrosis, bronchiectasis, chronic obstructive pulmonary disease, primary ciliary dyskinesia, and interstitial lung disease) [1–4]. In contrast to parenteral and oral formulations, inhaled antibiotics offer the distinct advantage of delivering high concentrations of drug to the site of infection while limiting systemic exposure. However, inhaled antibiotics may cause side effects, such as cough, dysphonia, bronchospasm, throat irritation, dyspnea, wheezing, and oropharyngeal pain. In published clinical trials, inhaled antibiotics have also been reported to potentially exacerbate preexisting pulmonary symptoms [1, 5–11].

For patients with nontuberculous mycobacterial (NTM) lung disease caused by *Mycobacterium avium* complex (MAC), guidelines recommend initial therapy with a multidrug regimen comprising a macrolide (clarithromycin or azithromycin), ethambutol, and a rifamycin (rifabutin or rifampin) until sputum culture is negative for 12 months [12, 13]. For patients with refractory disease, intensification of antibiotic treatment may be warranted [12, 13]. Amikacin liposome inhalation suspension (ALIS; ARIKAYCE) is a nebulized formulation of amikacin approved by the US Food and Drug Administration (FDA) as part of combination therapy for adults with treatment-refractory MAC lung disease with limited or no alternative treatment options [14]. It is important to note that until the approval of ALIS in September 2018, there were no FDA-approved treatments for refractory MAC lung disease.

In the phase 3 CONVERT study, respiratory adverse events of mild or moderate severity were reported in 87% of patients receiving ALIS in addition to guideline-based therapy (GBT) vs 50% of those receiving GBT alone [10]. Respiratory adverse events included both upper airway (dysphonia, cough, oropharyngeal pain) and lower respiratory tract events (dyspnea, wheezing, bronchospasm) and tended to have an onset within the first month of treatment. Dysphonia was the most commonly reported adverse event in the phase 2 study (43% of patients receiving ALIS vs 9% receiving placebo) [11] and in the phase 3 CONVERT study (47% of patients receiving ALIS plus GBT vs 1% receiving GBT alone) [10]. In clinical practice, failure to adequately manage respiratory adverse events associated with inhaled antibiotics, including ALIS, may lead to poor treatment adherence and early discontinuation of an important therapeutic modality.

We report patient perspectives obtained via a patient-directed survey regarding the techniques and strategies used in our clinics to manage respiratory adverse events associated with ALIS. This report is intended to provide insight into the management of these respiratory adverse events as practiced by patients and providers.

## SURVEY METHODS

All patients who were prescribed ALIS at 2 academic medical centers in the United States (Emory University, Atlanta, GA, USA, and Louisiana State University Health Sciences Center Clinics, New Orleans, LA, USA) were invited to participate in a survey to assess the incidence, frequency, and type of respiratory adverse events that occurred with initiation of ALIS, as well as the effectiveness of physician-directed strategies to manage the events. The survey was administered by each clinic’s staff from August through September 2019. Institutional review board approval was obtained at each site before survey administration.

The telephone survey comprised yes/no and open-ended questions and a 4-point Likert scale to evaluate dysphonia (“hoarseness”), cough, increased sputum production, and dyspnea (“shortness of breath”) (Supplementary Data). Data were analyzed using descriptive statistics, and trends in management strategies were identified from open-ended responses.

## SURVEY RESULTS

A total of 26 of 33 ALIS-treated adults with refractory MAC lung disease completed the survey (Emory University, n = 12; Louisiana State University Health Sciences Center, n = 14). Most had nodular bronchiectasis (73.1% [n = 19]) with or without other comorbid noncavitary pulmonary conditions (eg, chronic obstructive pulmonary disease [COPD], asthma); 26.9% (n = 7) had cavitary disease. Most respondents (92.3% [n = 24]) reported experiencing ≥1 adverse event category included in the survey; 30.7% (n = 8) of respondents reported experiencing 1 or 2 of these adverse event categories, and 61.5% (n = 16) reported experiencing adverse events in 3 or 4 categories. There were 4 patients who discontinued ALIS treatment due to severe adverse events (1 each with hemoptysis and hearing loss and 2 with hypersensitivity pneumonitis). It is our practice and recommendation to discontinue any medication that causes a severe adverse event, such as hypersensitivity pneumonitis or hearing loss.

Of the 24 patients who experienced respiratory adverse events after initiation of ALIS and reported them in the survey, 79.2% (n = 19) had ≥1 adverse event that needed management; all 19 of these patients reported improvement in ≥1 adverse event category following implementation of a management strategy. A summary of physician-directed management strategies and outcomes based on the survey is shown in Table 1.

**Table 1.** Respiratory Adverse Event Management Strategies and Outcomes

Adverse Event	Patients who Reported an Adverse Event, n/N (%)	How Often Adverse Events Are Experienced, n/N (%)	Patients who Required Management for Adverse Events, n/N (%)	Management Strategies	Patients who Improved With Management, n/N (%)
Dysphonia	19/26 (73.1)	Sometimes: 5/19 (26.3)Most of the time: 10/19 (52.6)	13/19 (68.4)	Symptomatic: antitussives, lozenges, warm water or glycerin gargle, rinsing mouth after nebulizer use Temporary dosing adjustment: change ALIS administration to the evening or temporarily reduce frequency of ALIS dosing	11/13 (84.6)
Increased sputum	18/26 (69.2)	Sometimes: 8/18 (44.4)Most of the time: 7/18 (38.9)	4/18 (22.2)	Airway clearance: airway clearance/ pulmonary hygiene	3/4 (75.0)
Increased cough	18/26 (69.2)	Sometimes: 8/18 (44.4)Most of the time: 8/18 (44.4)	11/18 (61.1)	Pharmacological: bronchodilator pretreatment Temporary dosing adjustment: temporary reduction in frequency of ALIS dosing or brief interruption of treatment	8/11 (72.7)
Dyspnea	15/26 (57.7)	Sometimes: 5/15 (33.3)Most of the time: 6/15 (40.0)	11/15 (73.3)	Pharmacological: bronchodilator pretreatment Temporary dosing adjustment: temporary reduction in frequency of ALIS dosing or brief interruption of treatment	10/11 (90.9)

Abbreviation: ALIS, amikacin liposome inhalation suspension.

### Dysphonia

Dysphonia was reported by 73.1% of patients (n = 19), with most (n = 13) needing management of this adverse event. Strategies for managing dysphonia included symptomatic management (eg, lozenges, n = 6; warm water or glycerin gargle, n = 8; antitussive agents, n = 1; changing ALIS administration to the evening, n = 5; temporarily reducing dosing frequency or briefly interrupting treatment, n = 4). Overall, intervention was effective at improving symptoms in 84.6% of patients (n = 11) with dysphonia who implemented a management plan. Three patients reported improvement in dysphonia without intervention.

### Increased Sputum Production

Increased sputum production was reported by 69.2% of patients (n = 18) and was sometimes associated with drug administration (during or soon after ALIS inhalation). Most patients did not need intervention to manage the increase in sputum production. Airway clearance improved symptoms in 3 of 4 patients. Additionally, patients were counseled that increased sputum production was an expected outcome with most nebulized antibiotics and may be a form of airway clearance in itself.

### Increased Cough

Increased cough was reported by 69.2% (n = 18) of patients and was also associated with drug administration. Of 11 patients who needed to manage their cough symptoms, use of a bronchodilator (n = 5; 3 patients improved) and temporary reduction in frequency or brief interruptions of ALIS (n = 4; 4 patients improved) were the most effective management strategies. Symptomatic management alone (eg, cough lozenges, n = 1; antitussive agents, n = 1) was the least effective method of improving cough. Overall, cough improved in 72.7% of patients (n = 8/11) who implemented a management strategy. Two patients reported improvement in cough without intervention.

### Dyspnea

Dyspnea was reported by 57.7% of patients (n = 15), of whom 73.3% (n = 11) implemented a management strategy. The most common management strategy for dyspnea was bronchodilator use (n = 7) and/or temporary reduction in frequency or brief interruptions of ALIS (n = 2). One patient who did not want to use a bronchodilator managed shortness of breath by limiting physical activity. One patient increased oxygen intake. With intervention, most patients with dyspnea (n = 10/11) improved. One patient with asthma, allergic bronchopulmonary aspergillosis, and bronchiectasis did not improve with albuterol use. One patient reported some improvement in dyspnea without intervention.

## DISCUSSION

### Optimizing Respiratory Adverse Event Management

MAC lung disease is becoming increasingly prevalent globally and is particularly common in older adults with underlying chronic lung disease [15]. ALIS is an add-on treatment option for patients with MAC lung disease for whom treatment with guideline-based therapy has been unsuccessful. Not unlike the results from the ALIS phase 2 and 3 clinical trials, the results from this patient-directed survey indicate that in our clinics most patients who were taking ALIS as part of their MAC lung disease treatment regimen experienced respiratory adverse events; however, the applied management techniques communicated to and practiced by our patients were generally effective in improving the common respiratory adverse events of dysphonia, increased sputum production, increased cough, and dyspnea.

### Symptom Management

Local adverse effects of inhaled drugs on the upper airways, including dysphonia [1], were managed through intake of soothing fluids, gargling with warm water or glycerin postdosing, and rinsing the mouth after completion of nebulizer treatment. Additionally, changing the time of ALIS administration to the evening was an effective management strategy. In our clinics, these strategies were successfully used to manage dysphonia and (to a lesser extent) cough associated with ALIS treatment. It should be noted that corticosteroids, either systemic or inhaled, were not used in the management of respiratory adverse events in this patient population, as emerging evidence suggests an association between corticosteroid use and NTM pulmonary infection [16, 17].

### Airway Clearance

Both underlying respiratory disease and the potential sputum mobilization effects of treatments such as ALIS may contribute to significant mucus production [12, 18]. Published reviews on the optimization of airway clearance—including specific breathing techniques (eg, active cycle of breathing, huff cough, postural positioning, autogenic drainage), chest percussion, and positive expiratory pressure therapy—suggest that these techniques may also improve symptoms in patients with serious lung infections such as MAC lung disease [18–20]. Nebulized hypertonic saline has been reported to facilitate mucociliary clearance [20]; if implemented in patients taking ALIS, they should be educated that the ALIS-specific LAMIRA nebulizer (PARI Pharma GmbH) is not FDA approved for this purpose. Airway clearance strategies should be customized to individual patients to account for their preferences, abilities, motivation, and underlying lung disease.

### Bronchodilator Use

Pretreatment with a short-acting bronchodilator before ALIS treatment in patients with a history of bronchospasm may be appropriate [14]. In our survey, patients reported that bronchodilator use was effective in improving cough and dyspnea. In our opinion, patients with preexisting hyperreactivity or COPD may benefit from optimization of background treatment with a long-acting inhaled bronchodilator and, if needed, could also use a short-acting bronchodilator before ALIS treatment.

### Temporary Changes in ALIS Dosing Schedule

Similar to the CONVERT study [10], in which investigators could temporarily interrupt ALIS dosing if patients experienced distressing local respiratory events, in our clinics, short-term suspension of ALIS (up to a maximum of 14 days) and temporary reduction in dosing frequency to 3 days a week until adverse events resolved were effective management strategies for patients experiencing increased cough, dyspnea, or dysphonia. In our experience, these adverse events did not recur or were mild after a brief ALIS interruption.

## CONCLUSIONS

ALIS is the only medication for refractory MAC lung disease that is FDA approved. Results from our patient-directed survey were consistent with the findings from the CONVERT trial in that patients treated with ALIS commonly experienced respiratory adverse events that were not treatment limiting. The survey results presented here indicate that respiratory adverse events commonly occurring with ALIS use can be managed with a variety of techniques and over-the-counter approaches to allow patients continued use of this treatment. Our collective clinical experience further reinforces that education of both patients and their extended care team—including nursing staff and respiratory therapists—may aid in early recognition and management of respiratory adverse events and contribute to both tolerability and adherence. Regardless of the management techniques chosen, patient education before and during ALIS treatment is essential for optimizing the potential for successful therapy. Well-informed patients who are more aware of potential treatment-related adverse events and possible management strategies may be more likely to contact their health care providers for guidance and/or initiation of symptom management approaches without necessarily needing provider intervention.
